# Relationship Between Systemic Inflammation and Glycemic Control in Firefighters

**DOI:** 10.3390/jfmk10020148

**Published:** 2025-04-27

**Authors:** Tiffany J. Oberther, Andrew R. Moore, Austin A. Kohler, A. Maleah Holland-Winkler

**Affiliations:** Department of Kinesiology, Augusta University, Augusta, GA 30909, USAandmoore@augusta.edu (A.R.M.); akohler@augusta.edu (A.A.K.)

**Keywords:** inflammation, diabetes, insulin resistance, HOMA-IR, C-reactive protein, tactical athletes, homocysteine, HbA1c

## Abstract

**Background**: Firefighters are at risk for developing metabolic diseases such as type 2 diabetes due to occupational-related stress and poor health behaviors. Firefighters often experience chronic inflammatory responses that may contribute to the development of insulin resistance. This study examined the relationship between systemic inflammation markers and glycemic control markers in firefighters. **Methods**: Blood samples were collected from twenty full-time male firefighters to assess HbA1c, fasting glucose, and insulin to estimate the Homeostatic Model of Assessment of Insulin Resistance (HOMA-IR), C-reactive protein (CRP), and homocysteine. Body composition and cardiovascular metrics were also recorded. Pearson partial correlation analyses were performed to evaluate relationships between homocysteine and CRP and the variables HOMA-IR and HbA1c while controlling for age and body fat percentage (BF%). SPSS version 29 was used for all analyses (α = 0.05). Data transformation was used where appropriate to ensure the normal distribution of each variable. **Results**: A significant positive correlation was found between homocysteine and HbA1c before (*p* = 0.006, *r* = 0.605) and after controlling for age and BF% (*p_partial_* = 0.013, *r_partial_* = 0.588), indicating that homocysteine levels are associated with impaired glycemic control in firefighters. No other relationships were found to be significant. **Conclusions**: The findings support a potential link between systemic inflammation and poor glycemic control in firefighters. Due to the occupational hazards that contribute to chronic inflammation, targeted interventions such as dietary modifications may help decrease the risk of diabetes and cardiovascular disease in this high-risk population.

## 1. Introduction

About 537 million instances of diabetes have been reported worldwide, with type 2 diabetes accounting for about 90% of these cases [1]. The prevalence of type 2 diabetes is still rising worldwide due to the rise in obesity-related sedentary lifestyles and bad eating habits [2]. Type 2 diabetes is a complicated, cardiorenal–metabolic disease entity that is brought on by consistently high blood glucose levels [1]. Multi-organ insulin resistance and a reduction in beta cell insulin secretory function are the causes of this condition [3].

Firefighters frequently operate in physically and psychologically challenging environments while playing a vital role in preserving lives and property. But according to recent research, firefighters are more likely than members of other professional groups to develop type 2 diabetes, even though they lead an active lifestyle [4]. Numerous environmental and occupational factors, such as high stress levels, disturbed sleep cycles, and obesogenic behaviors, including irregular eating habits, are blamed for this elevated risk [5]. These illnesses increase the risk of insulin resistance and diabetes by causing metabolic abnormalities [4]. Furthermore, diabetes might affect a firefighter’s physical skills and response time during emergencies, as firefighters must maintain optimal physical performance to carry out their jobs safely [6].

The Homeostatic Model Assessment for Insulin Resistance (HOMA-IR) and glycated hemoglobin (HbA1c) are two essential biomarkers for diabetes management. Average blood glucose levels during the two or three months prior to testing are reflected in HbA1c, which offers a trustworthy indicator of long-term glycemic control [7]. Elevated HbA1c values in firefighters are cause for concern, as they indicate inadequate glucose management, which may impair their physical capabilities during demanding tasks. However, insulin resistance, a crucial element in the development of type 2 diabetes from metabolic disorders, is measured by the HOMA-IR [8]. Increased HOMA-IR levels suggest a reduced capacity to use insulin efficiently, putting firemen at risk for consequences from diabetes, including cardiovascular disease [9].

In addition to diabetes, systemic inflammation is another condition to which firefighters are especially vulnerable. Inflammatory reactions that may continue beyond firefighting operations are caused by exposure to heat, smoke, and air pollutants during fire suppression activities [10]. Firefighters have been found to have elevated levels of inflammatory markers, including interleukin-6 (IL-6) and C-reactive protein (CRP), which are associated with an increased risk of metabolic and cardiovascular disorders [10,11,12]. Despite their initial protective effects, these inflammatory reactions raise the likelihood of long-term health issues like insulin resistance and type 2 diabetes [13].

Homocysteine and C-reactive protein (CRP) are two significant inflammatory markers that offer information on the degree of systemic inflammation. Increased oxidative stress and endothelial dysfunction, two major causes of cardiovascular disease, have been linked to elevated homocysteine [14]. Likewise, CRP, an acute-phase reactant, has been connected to the onset of diabetes and cardiovascular problems and increases in response to inflammatory stimuli [15]. Firefighters with elevated CRP have chronic inflammation, which worsens insulin resistance and raises the risk of abrupt cardiac events [16,17,18].

It is widely acknowledged that inflammation and diabetes are related to one another. It has been demonstrated that persistent low-grade inflammation disrupts insulin signaling and encourages β-cell malfunction, two important pathways in the etiology of type 2 diabetes [19]. A feedback loop that prolongs metabolic dysfunction and makes managing diabetes more difficult is created when proinflammatory cytokines like IL-6 and CRP exacerbate insulin resistance [20].

This study aimed to determine the correlation between diabetic and inflammatory markers in firefighters. Prior studies have found that the related medical conditions of inflammation and diabetes pose a significant obstacle for firefighters. Controlling these factors is essential for the safety and effectiveness of firefighting operations and the health of each firefighter. Targeted therapies that address the dual dangers of diabetes and inflammation can improve firefighters’ health and keep them fit for duty with the ability to respond to emergencies.

## 2. Materials and Methods

### 2.1. Study Design

A cross-sectional design was used to determine the relationship between diabetic and inflammatory markers in full-time firefighters in the southeastern part of the United States. All participants had their blood drawn during the same week in July 2024 to assess diabetic and inflammatory markers. Body composition and cardiovascular characteristics were measured in a separate laboratory visit within two weeks of the blood draw. This study was approved by the University’s Institutional Review Board (IRBNet ID: 2095651), all procedures performed followed institutional guidelines, and all participants signed an informed consent.

### 2.2. Participants

Twenty full-time male firefighters (39.4 ± 12.5 years old) participated in this study. All were employed by the same fire department, which consists of 16 different stations. While both males and females were eligible for inclusion, only males volunteered, as they represent over 98% of the department. Participants were excluded if they were (1) pregnant or (2) using vasoactive medications, including catecholamines, phosphodiesterase inhibitors, calcium sensitizers, or vasopressors.

### 2.3. Protocol

During the initial meeting, the participants provided a list of their medications to verify eligibility based on the inclusion criteria. They then signed an informed consent form to participate in the study. Preparation instructions were given for the blood draw and the body composition and cardiovascular health assessment visits, which were scheduled to take place within two weeks of each other.

#### 2.3.1. Body Composition and Cardiovascular Health Assessment

The preparatory instructions for body composition and cardiovascular health assessment included fasting with only water and abstaining from nicotine and antioxidant vitamins for 12 h, alcohol for 24 h, and moderate-to-vigorous intensity exercise for 8 h prior to the visit.

Height was measured with shoes off via a wall stadiometer (Stadi-o-meter; Novel Products, Inc., Rockton, IL, USA). A bioelectrical impedance scale (InBody 580 Body Composition Analyzer, Cerritos, CA, USA) was used to measure weight, BMI, and body fat percentage (BF%).

Cardiovascular health assessments were taken in the morning with a clinical multifunctional vascular testing system (PWV; Vicorder, 80 Beats Medical GmbH, Kantstrasse, Berlin, Germany) after participants rested in the supine position for 10 min. The cardiovascular health assessments included two vascular tests: pulse wave analysis and pulse wave velocity.

After resting for 10 min in the supine position, the participants continued to rest for the pulse wave analysis test. A pressure cuff was placed around the brachial artery and connected to the vascular testing system. The test provided measures of resting systolic and diastolic blood pressure, mean arterial pressure, pulse pressure, heart rate, and stroke volume.

After the pulse wave analysis test, the participants continued to rest in the supine position, and their shoulders were raised by 30 degrees for the aortic pulse wave velocity test. A neck pad was placed around the neck with a pressure pad over the right carotid area, secured with a Velcro fixing that was not too tight. A pressure cuff was placed around the right thigh and connected to the vascular testing system. The test provided a measure of pulse wave velocity indicative of arterial stiffness.

#### 2.3.2. Venous Blood Measure

Circulating diabetic and inflammatory markers were evaluated, including hemoglobin A1c (HbA1c), fasting glucose, insulin (to estimate HOMA-IR using the equation fasting glucose*insulin/405), circulating C-reactive protein (CRP), and homocysteine. The participants were instructed to fast for 8 h before the assessment, with only water permitted during this time.

Blood samples were collected from subjects via venipuncture using serum-separating tubes (SST). Following collection, the tubes were allowed to clot for 30 min at room temperature and then centrifuged at 1500× *g* for 10 min to separate the serum. The resulting serum was carefully divided to avoid contamination from cellular material or the gel separator. Serum samples were stored at 4 °C until analysis and processed within 24 h to maintain sample integrity.

Each serum sample was pipetted directly into standardized cuvettes for the IR 1200 calorimetric analysis, ensuring consistent sample volumes across all measurements.

##### HbA1c Analysis

The determination of hemoglobin A1c (HbA1c) levels was performed using the Diazyme Direct Enzymatic HbA1c Assay (Diazyme Laboratories, Poway, CA, USA). This method utilizes an enzymatic approach for the quantitative measurement of HbA1c in the whole blood samples, designed to provide reliable glycemic control monitoring. The Diazyme Direct Enzymatic HbA1c Assay was performed on an automated clinical chemistry analyzer. The HbA1c concentration was calculated using a calibration curve established with the provided calibrators. Quality control was maintained by testing two levels of HbA1c controls in each analytical run. The controls were provided in liquid or lyophilized form and stored at 2–8 °C after preparation.

##### Glucose Analysis

Blood glucose concentrations were quantified using a Synermed Glucose IR070 reagent analyzer (Infrared Laboratory Systems, LLC, Westfield, IN, USA) on a spectrophotometer, employing an enzymatic glucose oxidase methodology for the determination of glucose levels in serum or plasma samples. The assay was performed according to the manufacturer’s protocol. The absorbance reading, proportional to the glucose concentration, was compared to a calibration curve prepared using the Synermed glucose calibrator (300 mg/dL glucose). A protein-based reference material containing 300 mg/dL glucose was used for calibration, and calibration was performed daily prior to sample analysis. Quality control was ensured by testing normal and abnormal control materials, with acceptable ranges established by individual laboratories.

##### Insulin Analysis

The quantitative measurement of insulin in serum or heparinized plasma was performed using the IMMULITE 1000 Insulin Assay (Siemens Healthineers, Cary, NC, USA). Following the manufacturer’s protocol, this method employed a solid-phase, enzyme-labeled chemiluminescent immunometric assay to detect and analyze insulin. The assay was calibrated using insulin adjustors, and calibration was performed as recommended by the manufacturer. Insulin controls were used to ensure assay accuracy and precision. Quality control samples were run in every assay batch, and results falling outside acceptable ranges triggered troubleshooting or recalibration.

##### CRP Analysis

The quantification of CRP was performed using a Diazyme High Sensitivity CRP (hsCRP) Assay kit (Diazyme Laboratories, Poway, CA, USA), which employs an enhanced immunoturbidimetric assay methodology for the in vitro determination of CRP concentrations in human serum and plasma samples. The assay was conducted on an automated clinical chemistry analyzer, following the manufacturer’s instructions. The Diazyme hsCRP Calibrator set, consisting of four levels of CRP material, was utilized to establish the calibration curve. Calibration was performed every 14 days or as recommended by the manufacturer. Quality control was ensured using normal and abnormal CRP control materials, and control results were required to fall within acceptable ranges established by individual laboratories.

##### Homocysteine Analysis

The quantification of total L-homocysteine (Hcy) in serum and plasma was performed using a Diazyme Homocysteine 2 Reagent Enzymatic Assay kit (Diazyme Laboratories, Poway, CA, USA). The assay is based on an enzymatic conversion and co-substrate cycling system, allowing for the accurate determination of Hcy concentrations using an automated clinical chemistry analyzer equipped with absorbance measurement capabilities at 340 nm. The assay was calibrated using five levels of Hcy calibrator material. Calibration was performed at least every 5 days. Quality control was ensured by testing a set of normal and abnormal control materials. Laboratory-specific acceptable ranges were established for these controls.

### 2.4. Statistical Analysis

Separate Pearson partial correlation analyses were used to determine the relationships between markers of systemic inflammation (CRP and homocysteine) and indicators of diabetes [HOMA-IR and Hemoglobin A1c (HbA1c)] while controlling for BF% and age (in years), which are related to systemic inflammation [21]. CRP and homocysteine were treated as predictor variables, and HOMA-IR and HbA1c were treated as outcome variables. SPSS version 29 was used for all analyses (α = 0.05). Data for each variable were screened for missing values and univariate outliers, defined as scores with a standardized value ≥ 3.0 standard deviation units from the group mean (Z ≥ 3.0). The results of the Shapiro–Wilk test were interpreted to determine if each variable was normally distributed (*p* > 0.05). Violation of the assumption of normal distribution required a transformation procedure as appropriate so that planned parametric correlation analyses could proceed. The linear relationship between variables was verified using a visual interpretation of scatterplots with Loess lines. Data were screened for multivariate outliers defined as a Mahalanobis D value greater than the critical value of 18.47 (based on 4 variables per partial correlation analysis and an alpha level of 0.001). The correlation coefficient Pearson’s *r* (*r*) ranged from −1.0 to 1.0 and was interpreted according to direction (i.e., positive = direct relationship between variables; negative = indirect relationship between variables) and magnitude (0–0.3 = weak relationship; 0.31–0.5 = moderate relationship; 0.51–1.0 = strong relationship).

## 3. Results

Cardiovascular and body composition characteristics are included in Table 1.

Univariate outliers (≥3.0 SD from the group mean) were detected for the variables CRP (1 outlier—6.30; Z = 3.19), homocysteine (1 outlier—26.79; Z = 3.02), and HOMA-IR (1 outlier—6.97; Z = 3.09). These variables were removed from the respective correlation analyses via pairwise deletion. The analyses differ in terms of sample size for this reason. No multivariate outliers were detected. Descriptive statistics for each variable following the removal of outliers are presented in Table 2.

The variables HOMA-IR, HbA1c, and CRP violated the assumption of normality. All variables were transformed using the natural logarithm procedure [22], after which a normal distribution for the data for each variable was met. For variables with data values between 0 and 1, a constant was added to all scores so that the smallest value was 1.0 before logarithmic transformation [23].

The data were analyzed before and after transformation to better interpret the effect of data transformation on the relationships between variables of interest. For each version of the data (non-transformed and transformed), *r*-values and *p*-values are presented for zero-order correlations between variables (i.e., without controlling for BF% and age) and partial correlations between variables (*r_partial_* and *p_partial_*; i.e., controlling for BF% and age). All statistical results are presented in Table 3 and summarized briefly in the text. Among the non-transformed (raw) scores, there was a strong, positive, and significant relationship between homocysteine and HbA1c. This relationship was still present after controlling for age and body fat percentage. The unstandardized beta correlation coefficient in this case was 0.10, which indicates that each 10 μmol/L increase in homocysteine is associated with a 1% increase in HbA1c. Transforming the scores to satisfy the assumption of a normal distribution did not substantially change the characteristics of the relationship between homocysteine and HbA1c. Finally, this relationship was still present after statistically controlling for age and body fat percentage in the analysis of the transformed scores. All of the remaining relationships investigated were not significant and ranged in magnitude from small to moderate.

**Figure 1:**
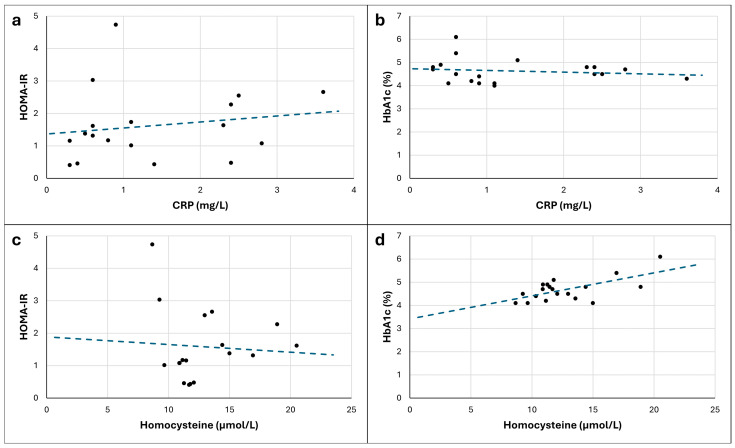
Scatterplots showing the visual representation of the relationships between the non-transformed version of variables: (**a**) C-reactive protein (CRP) and Homeostatic Model Assessment of Insulin Resistance (HOMA-IR); (**b**) CRP and glycated hemoglobin (HbA1c); (**c**) homocysteine and HOMA-IR; and (**d**) homocysteine and HbA1c. The blue dashed line is the trendline that corresponds to the data points of the individuals plotted as black dots.

Given that the results of the partial correlation analyses were not substantially or significantly different between the non-transformed and transformed versions of the scores, the visual depiction of the non-transformed scores are presented to increase the interpretability of the relationships between variables. These scatterplots are presented in Figure 1.

## 4. Discussion

The findings of this study indicate a significant direct relationship between homocysteine and HbA1c levels among firefighters, even after adjusting for age and body fat percentage. This suggests that elevated homocysteine levels may contribute to poor glycemic control in this occupational group, thereby increasing their risk for diabetes and cardiovascular disease (CVD). For example, our results suggest that a 10 μmol/L increase in homocysteine is associated with a 1% increase in HbA1c, a hypothetical difference which would classify half of the 20 participants as having diabetes (HbA1c ≥ 6.5%) or pre-diabetes (5.7% < HbA1c < 6.5%) [1]. Given firefighting’s strenuous and hazardous nature, understanding these metabolic relationships is crucial for developing targeted interventions.

Several studies support our findings of a direct correlation between homocysteine and HbA1c. Noor et al. conducted a study on 188 patients with type 2 diabetes and demonstrated that over 80% of the individuals had poor glycemic control (HbA1c > 7%). They found a positive correlation between HbA1c and serum homocysteine levels, with severe hyperhomocysteinemia in over 80% of the patients with an HbA1c greater than 7% [24]. Similarly, Kotchapetch et al. demonstrated a significant relationship between HbA1C and CRP in adults with pre-diabetes or diabetes [25]. They also found relationships between CRP, homocysteine, and BMI. While many studies confirm this association, others have failed to establish a significant link between hyperhomocysteinemia and glycemic control, suggesting that additional factors, such as inflammation and oxidative stress, may also play a role [24,25,26]. For instance, Zulfania et al. found no relationship between homocysteine and HbA1c or BMI in 125 patients with type 2 diabetes. However, homocysteine in this patient population was found to be related to systolic blood pressure [27].

Beyond its relationship with HbA1c, homocysteine has been linked to hypertension. Our study population demonstrated high systolic blood pressure (132.2 mmHg) and pulse pressure (62.3 mmHg) on average, conditions that have been associated with elevated homocysteine levels in other studies and contribute to greater cardiovascular risk [28,29]. However, the Framingham Heart Study, which included 2104 individuals without hypertension at baseline, failed to establish a causal relationship between homocysteine and hypertension, underscoring the complexity of these interactions [30].

To determine if body composition impacted the relationship between glycemic control and inflammatory markers, we demonstrated the results for controlling and not controlling for body fat percentage. Since our population may possess more muscle than the average population due to the physical nature of their occupation, we chose to analyze body fat percentage instead of BMI. Our results revealed that controlling for body fat percentage did not alter the overall declaration of significance between the variables. However, we did not assess the direct relationship between the firefighters’ body fat percentage and glycemic control or inflammatory markers. Pettersson-Pablo et al. showed a relationship between body fat percentage and inflammatory markers, including CRP, in healthy young adults [31]. Garcia-Rubira et al. explained the mechanisms linking obesity to the activation of inflammation primarily through the NLRP3 inflammasome. Since body fat may induce a proinflammatory state, controlling for body fat or BMI is imperative when assessing other markers related to inflammation [32].

Firefighters may be particularly susceptible to elevated homocysteine levels due to the inflammatory nature of their occupation. Exposure to fire emissions, including hazardous pollutants, can lead to acute and chronic systemic inflammation, which may elevate inflammatory markers, such as homocysteine levels [5]. Long-term firefighting careers have been associated with prolonged systemic inflammation, increasing the risk of cardiovascular and metabolic diseases [33,34]. Additionally, mild hyperhomocysteinemia is a known independent predictor of CVD, and firefighters’ increased vascular risk may be partially attributed to this metabolic disturbance [35,36].

To reduce inflammation and ultimately lower the risk of diabetes and cardiovascular disease (CVD) in firefighters, several practical interventions can be implemented. Strategies such as increasing dietary folate intake through supplementation or fortified foods have been shown to lower homocysteine concentrations effectively [37,38,39,40]. Furthermore, structured self-monitoring blood glucose interventions focusing on lowering HbA1c levels and improving glycemic control may also reduce systemic inflammation, as evidenced by reductions in high-sensitivity C-reactive protein (hs-CRP) levels [41]. Additionally, lifestyle modifications, including regular physical activity, a balanced diet, and stress management, can help mitigate inflammation and reduce the risk of chronic diseases in firefighters [42,43].

One limitation of this study includes the relatively small sample size of 20 participants. Correlation analyses with a sample size below 30 are highly sensitive to the influence of outliers, which could lead to conclusions that are not generalizable to the greater population [44]. Although we screened the data for outliers and transformed the data to yield a normally distributed set of scores, a larger sample would be more desirable to draw stronger conclusions about the relationships between inflammation and insulin resistance in this population. A second limitation of this study is the somewhat restricted range of the variables encountered in this generally healthy sample. For example, HbA1c did not exceed 6.1%, suggesting that this sample exhibited healthy glycemic control. The generalizability of the findings presented here may not extend to firefighters with impaired glycemic control.

## 5. Conclusions

This study revealed a significant relationship between HbA1c and homocysteine, even after controlling for body fat percentage and age. In other words, a higher level of homocysteine was associated with poorer glycemic control regardless of age or body composition. No relationship was found between HOMA-IR and either inflammatory marker or HbA1c and CRP. Follow-up studies should address this issue by collecting data from larger samples of firefighters with more heterogeneous health and metabolic function levels. Additionally, future research should incorporate lifestyle questionnaires to gain insights into health behaviors contributing to inflammation and interventions specifically designed to reduce inflammation and its metabolic consequences in firefighters. Addressing these factors may ultimately lead to reduced diabetes and CVD risk in this high-risk occupational group.

## Figures and Tables

**Table 1:** Mean cardiovascular and body composition characteristics for the 20 male participants.

Resting Cardiovascular Characteristics	
Measure (Unit)	Mean ± Standard Deviation
Systolic Blood Pressure (mmHg)	132.2 ± 8.6
Diastolic Blood Pressure (mmHg)	70.6 ± 9.5
Pulse Pressure (mmHg)	62.3 ± 5.7
Mean Arterial Pressure (mmHg)	94.7 ± 8.8
Heart Rate (bpm)	57.4 ± 7.8
Estimate Stroke Volume (mL)	115.7 ± 21.1
Peripheral Resistance (PRU)	0.9 ± 0.2
Pulse Wave Velocity (m/s)	6.5 ± 0.9
**Body Composition Characteristics**	
Weight (lb)	220.8 ± 39.6
Height (in)	70.8 ± 2.8
BMI (kg/m^2^)	31.0 ± 5.2
Body Fat (%)	28.1 ± 7.3
Lean Body Mass (lb)	156.7 ± 19.4
Total Body Water (lb)	115.1 ± 14.0

**Table 2:** Descriptive statistics for the variables used in the formal statistical analysis.

Variable (Units)	Mean	Standard Deviation
CRP (mg/L)	1.342	0.998
Homocysteine (μmol/L)	12.698	1.083
HOMA-IR	1.592	1.083
HbA1c (%)	4.645	0.505

**Table 3:** Statistical results for zero-order and partial correlation between variables, before and after transformation.

	**Variables**	**Zero-Order Correlations**		**Partial Correlations**	
		*r*	*p*	*r_partial_*	*p_partial_*
Non-transformed scores	CRP				
	HOMA-IR	0.170	0.499	−0.024	0.929
	HbA1c	−0.144	0.556	−0.059	0.822
	Homocysteine				
	HOMA-IR	−0.069	0.787	−0.351	0.183
	HbA1c	0.643	0.003 *	0.632	0.007 *
	**Variables**	**Zero-Order Correlations**		**Partial Correlations**	
		*r*	*p*	*r_partial_*	*p_partial_*
Transformed scores	CRP				
	HOMA-IR	0.231	0.356	−0.063	0.818
	HbA1c	−0.150	0.541	−0.083	0.752
	Homocysteine				
	HOMA-IR	0.011	0.964	−0.305	0.250
	HbA1c	0.605	0.006 *	0.588	0.013 *

Notes: * = indicates a significant relationship. Zero-order correlations are the correlation analysis results without controlling for age and body fat percentage.

## Data Availability

The data collected and analyzed for this study are freely available at the online data repository Open Science Framework via the following web address: https://osf.io/js8ne/ (last updated 27 February 2025).
